# Beyond the bone flap: integrating biomarkers, imaging, and emerging technologies in the surgical continuum of acute subdural hematoma (ASDH)

**DOI:** 10.1097/MS9.0000000000005152

**Published:** 2026-05-14

**Authors:** Shreya Singh Beniwal, Rafael Everton Assunção Ribeiro da Costa, Chimuka Mwangaa, Elif Özge Çelik, Dawson Joshino Rebello, Prashasti Dahiya, Aarushi Mishra

**Affiliations:** a Lady Hardinge Medical College, New Delhi, India; b State University of Campinas (UNICAMP), Cidade Universitária “Zeferino Vaz”, Campinas, São Paulo, Brazil; c Texila American University Zambia, Lusaka, Zambia; d Van Provincial Health Directorate, Turkish Ministry of Health, Hatuniye, İpekyolu, Van, Türkiye; e Tbilisi State Medical University, Tbilisi, Georgia; f ESIC Medical College and Hospital, New Industrial Township, Faridabad, India; g Danylo Halytsky Lviv National Medical University, Lviv, Ukraine

**Keywords:** acute subdural hematoma, advanced neuroimaging, decompressive craniectomy, neurotrauma biomarkers, surgical decision-making

## Abstract

Acute subdural hematoma (ASDH) is a life-threatening neurosurgical emergency associated with high mortality and long-term functional disability. Despite advances in surgical techniques, outcomes remain variable, highlighting the need for a more integrated management approach. Traditional decision-making has focused primarily on choosing between craniotomy and decompressive craniectomy, without fully incorporating postoperative recovery and rehabilitation. A narrative synthesis of literature published between 2010 and 2025 was conducted using PubMed, Embase, and Scopus databases. Studies were identified using predefined keywords related to “acute subdural hematoma,” “traumatic brain injury,” “biomarkers,” “neuroimaging,” and “surgical management,” and were selected based on relevance to ASDH-specific or severe traumatic brain injury literature, with prioritization of high-quality clinical and translational studies. Advances in biomarkers, imaging modalities, surgical strategies, and emerging technologies were analyzed with emphasis on building a continuous care model for ASDH. Findings indicate that integrating inflammatory, coagulation, and neurodegeneration biomarkers enhances early risk stratification and supports surgical decision-making. The role of cranioplasty is no longer merely reconstructive but therapeutic, aiding in the restoration of cerebral hemodynamics, brain metabolism, and cognitive recovery. Emerging technologies – including magnetic resonance–guided focused ultrasound, 3D-printed implants, and AI-based imaging analytics – expand opportunities for personalized treatment. In conclusion, ASDH should be understood as a surgical-therapeutic continuum extending beyond hematoma evacuation, where the integration of biological insights, technological advances, and equity principles can transform survival, functional outcomes, and quality of life across diverse global healthcare settings.

## Introduction

Traumatic brain injury (TBI) represents a substantial global health burden, with millions of new cases each year contributing to significant mortality and long-term disability**^[^1,2^]^**. Recent Global Burden of Disease estimates suggest that approximately 20.8 million people sustain TBI annually, although broader data, including mild cases, suggest this number may be as high as 55–60 million**^[^2,3^]^**. These differences reflect both evolving methodologies and the inclusion of all severities of injury, highlighting the challenges of measuring TBI’s true global scope. What remains clear, however, is that TBI exerts a profound and growing impact on patients, families, and healthcare systems worldwide.

HIGHLIGHTS

- ASDH is reframed as a continuous surgical and therapeutic care pathway.
- Biomarkers improve early risk stratification and surgical decision-making.
- Advanced imaging enhances prediction of edema, ischemia, and outcomes.
- Surgical strategies integrate craniotomy, decompression, and cranioplasty.
- Emerging technologies enable personalized and globally relevant ASDH care.

The burden of acute subdural hematoma (ASDH), one of the most devastating forms of TBI, is not fully captured by disability-adjusted life years (DALYs) alone**^[^3^]^**. Hospital admissions for ASDH continue to rise, largely due to aging populations, widespread use of antithrombotic medications, and improved neuroimaging leading to greater detection**^[^4^]^**. This underscores that epidemiological metrics may underestimate the real clinical workload associated with ASDH.

Surgical management of ASDH remains a central challenge. Craniotomy (CO) and decompressive craniectomy (DC) are the two dominant procedures, but their relative value continues to be debated**^[^5,6^]^**. Results from the RESCUE-ASDH trial provided important evidence, showing differences in mortality and functional outcomes between approaches, but also highlighted the specific risks of each procedure rather than a universal superiority**^[^5^]^**. While DC can reduce mortality, it carries risks of poor functional recovery and complications such as hydrocephalus or external brain herniation**^[^6^]^**. In contrast, CO is often preferred for its reconstructive advantages but may not be sufficient in cases of uncontrollable swelling. Together, these findings emphasize that surgical decisions should be individualized, guided by patient biology and physiology rather than a single-operation mindset**^[^7^]^**.

The challenges of ASDH management are especially pronounced in low- and middle-income countries (LMICs), where limited access to computed tomography (CT) scanning, delayed transfers, and shortages of intensive care unit beds severely constrain timely intervention**^[^1^]^**. Such inequities exacerbate the already heavy burden of disease and demand global efforts to strengthen neurosurgical capacity and training.

Looking forward, there is a need for a continuum-based framework that integrates biology, technology, and equity into decision-making. On the one hand, biology-driven approaches should account for individual patient factors such as age, frailty, and comorbidities. On the other hand, advances in imaging, monitoring, and surgical technology can refine operative strategies. Importantly, these innovations must be delivered equitably, ensuring that LMICs benefit alongside high-income regions**^[^7^]^**.

Ultimately, shifting ASDH care requires moving beyond the bone flap**^[^6^]^**. This means reframing surgical decision-making within a broader context of prevention, patient-centered outcomes, and global health equity. Such a transition will be critical to reducing the burden of ASDH and improving outcomes for patients worldwide.

The present manuscript was prepared in accordance with the Transparency in the Reporting of Artificial Intelligence in Research (TITAN) Guidelines 2025, governing transparency and responsible use of artificial intelligence (AI) in academic publishing**^[^8^]^**.

### Literature search strategy

This narrative review was based on a structured literature search conducted in the PubMed, Embase, and Scopus databases for studies published between 2010 and 2025. Keywords included combinations of “acute subdural hematoma,” “traumatic brain injury,” “biomarkers,” “radiomics,” “artificial intelligence,” “decompressive craniectomy,” and “craniotomy.” Studies were selected based on clinical relevance to ASDH or severe TBI, with an emphasis on original research, high-quality observational studies, and key translational evidence. Where ASDH-specific data were limited, carefully contextualized extrapolation from broader TBI literature was applied.

## Pathophysiological basis and surgical rationale

Many critical symptoms of ASDH are commonly caused by trauma and the accumulation of blood between the dura mater and the brain. This causes a dramatic increase in intracranial pressure (ICP), which is the main worry. Increased ICP leads to a decrease in cerebral perfusion pressure (CPP) and cerebral blood flow (CBF), which causes secondary ischemia through the loss of cerebral autoregulation and vasospasm**^[^9,10^]^**. The mass effect of the hematoma may, in turn, induce brain herniation, exacerbate ischemic insult to the brain, and result in poor outcomes **^[^10,11^]^**. Furthermore, brain edema exacerbates the condition by elevating pressure, causing more brain compression, and leading to irreparable tissue damage**^[^12^]^**.

The decision to perform DC or CO should take into consideration the degree of hematoma and underlying brain edema. DC is performed mainly in the presence of severe cerebral edema or when ICP cannot be controlled well, loosening a part of the skull to allow room for the swollen brain and reducing pressure and compression against the brain**^[^10,12^]^**. In CO, the hematoma is evacuated, and the bone flap is replaced. Less invasive than DC, CO may be ineffective in the setting of marked brain swelling because of limited room for brain expansion without additional damage**^[^9,10^]^**.

However, DC is far from perfect. One of the primary concerns is the inevitable requirement of cranioplasty – a second surgery requiring you to go under the knife for a second time post-braincare. Cranioplasty poses risks such as surgical site infection, bone resorption, and delayed healing**^[^12,13^]^**. However, DC is still effective in controlling ICP and avoiding additional neurological damage in patients suffering from severe continuous extraventricular drainage**^[^11^]^**.

### In the reversal of cranioplasty to regain cerebral compliance and perfusion

DC, followed by cranioplasty, leads to the reconstruction of the skull structure, a decrease in cerebral compliance, and an increase in the ease of brain perfusion. It also promotes “glymphatic flow,” which is critical to brain healing after surgery. However, the second operation is expected to increase the risk of infection, bone absorption, and recovery duration**^[^12,13^]^**. Nevertheless, cranioplasty is required to restore skull function and allow for the best possible perfusion of the brain after DC.

### Association of surgery and systemic inflammatory reaction

Both CO and DC induce systemic inflammation, which may exacerbate brain damage and delay recovery. Operative trauma-induced inflammation could contribute to complications, including infections, extended recovery, and neurologic deficits**^[^9,10^]^**. Cytokine release and oxidative stress after trauma and surgery can cause patients to have poor outcomes**^[^12^]^**. Thus, the modulation of these pro-inflammatory responses is important for enhancing the recovery rate and reducing complications**^[^11^]^**.

DC is generally considered for subgroups of patients who have severe cerebral edema or are at risk of ICP increase. Nevertheless, it is also linked to an increased risk of complications, such as wound infection and issues with cranioplasty, which may lead to prolonged recovery**^[^10,12^]^**.

CO might be more advantageous than DC in less severe brain swelling, where hematoma evacuation is more controllable without the secondary complexities that come with DC. On the other hand, however, CO carries a higher reoperation rate and an increased probability of ICP if cerebral swelling continues**^[^11,13^]^**.

When baseline patient severity is matched, DC and CO do not differ in terms of surgical outcomes, including mortality and functional recovery**^[^13^]^**. However, CO generally causes better functional recovery**^[^12^]^**.

In conclusion, DC and CO have their own merits, with the type of surgery being dependent on the degree of brain swelling, patient condition, and surgical skill. The use of DC can lead to superior ICP control in patients with significant cHL, while CO may be more suitable in the event of minimal edema. More clinical studies and real-world research are warranted to make decisions wisely in order to reduce the risks of surgery**^[^10,13^]^**.

## Molecular and genetic biomarkers in ASDH

While some evidence is derived specifically from ASDH cohorts, a substantial portion is extrapolated from broader moderate-to-severe TBI literature due to the limited availability of ASDH-exclusive studies. TBI triggers inflammation, blood clotting problems, and brain cell damage, which can be tracked with biomarkers. The presence of inflammatory, coagulation, and neurodegeneration biomarkers in the blood reflects blood–brain barrier disruption and may assist in risk stratification and prognostication; however, their direct role in guiding surgical decision-making remains investigational**^[^14–16^]^**.

### Inflammatory biomarkers

Mechanical injury activates microglia and astrocytes, leading to the release of cytokines such as IL-6 and CRP. IL-6 is a potential biomarker for clinical outcomes in patients after TBI. It is a protein that affects inflammation, nerve growth factor synthesis, and brain cell signaling**^[^14^]^**. Too much IL-6, however, worsens neuronal injury and swelling. Elevated levels of these cytokines in serum and cerebrospinal fluid (CSF) are associated with greater severity and worse outcomes, reflecting both local inflammation and systemic immune activation.

One study showed that surgical evacuation of hematomas reduced serum IL-6 postoperatively, highlighting the interplay between surgical timing and inflammation**^[^17^]^**. Clinically, patients with borderline CT findings but markedly elevated IL-6 and CRP are at higher risk of further damage. In such scenarios, consider a lower threshold for DC and aggressive neurocritical care monitoring.

### Coagulation biomarkers

ASDH disrupts balance through tissue factor release, endothelial injury, and fibrinolytic activation. This produces early abnormalities in coagulation and fibrinolysis, such as D-dimer elevation, fibrinogen reduction, and platelet dysfunction. Elevated D-dimer reflects ongoing clot breakdown**^[^15,18^]^**. These are common in acute head injury and predict hematoma expansion and worse neurologic outcomes **^[^15,19^]^**. Reduced fibrinogen and platelet dysfunction impair clot formation, further worsening intracranial bleeding. If left uncorrected, coagulopathy increases intraoperative bleeding risk and contributes to mass effect. In such cases, DC is often preferred for high-risk profile patients. Conversely, when coagulopathy is rapidly reversible with plasma, cryoprecipitate, or fibrinogen concentrate, CO is preferred for low-risk profile patients once hemostasis is achieved. Thus, coagulation markers may contribute to perioperative risk stratification and prognostication; however, their role in directly determining surgical strategy remains to be prospectively validated.

### Neurodegeneration biomarkers

**Figure 1.**
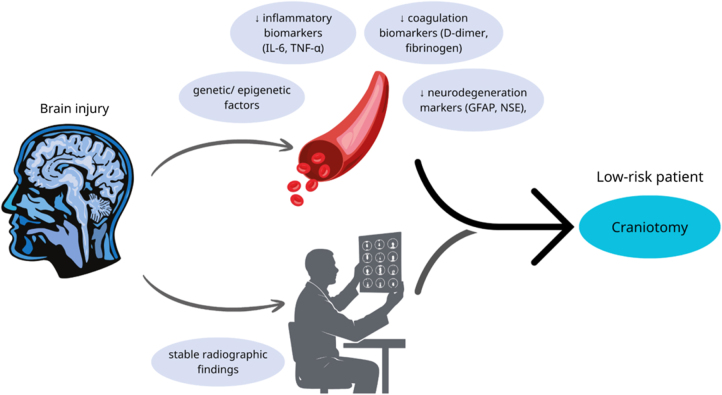
A schematic of injured brain showing low biomarkers in the blood plus stable CT findings, may support consideration of craniotomy (illustrative framework; not a validated clinical algorithm)**^[^14–16^]^**.

**Figure 2.**
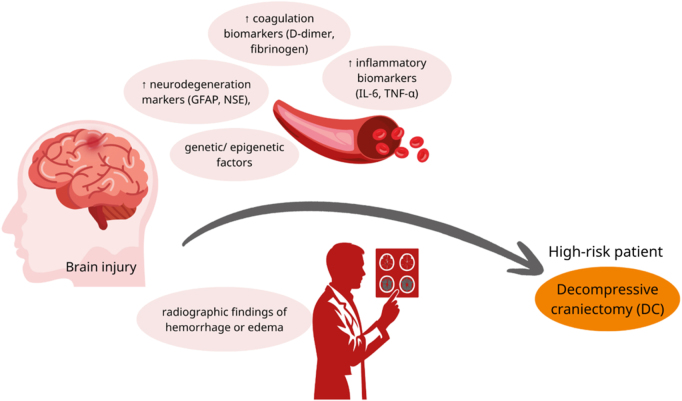
A schematic of injured brain showing elevated biomarkers plus poor radiographic findings (hemorrhage or edema), may support consideration of DC (illustrative framework; not a validated clinical algorithm)**^[^14–16,21^]^**.

Neuronal and glial damage release structural proteins that reflect parenchymal injury. NSE, derived from neurons, signals metabolic stress and cytoskeletal breakdown. GFAP, an astrocytic cytoskeletal protein, indicates glial injury in the central nervous system. Tau proteins and neurofilament-light (NF-L) represent axonal injury. These biomarkers identify patients at higher risk of hematoma expansion, coagulopathy, diffuse parenchymal injury, and refractory edema. Studies have demonstrated that elevation of these biomarkers is correlated with more severe tissue injury and worse outcomes**^[^15,20^]^**. GFAP and NF-L are the most significant predictors of TBI outcomes**^[^20^]^**. Therefore, they may augment current clinical and radiologic decision-making as adjunctive tools; however, their use in determining the choice between CO and DC remains investigational. As shown in Figure 1, patients with low biomarker risk combined with favorable CT findings may be candidates for CO**^[^14–16^]^**. Conversely, as shown in Figure 2, in patients with high biomarker risk with signs of swelling or mass effect on CT, consider DC**^[^14–16,21^]^**.

### Genomics and epigenetics

Genetic variability likely explains the clinical heterogeneity of cerebral edema formation in TBI. The ABCC8 (ATP-binding cassette sub-family C member 8) gene is a protein that encodes the sulfonylurea receptor 1 (SUR1). Differences in this gene affect ion channels and influence brain swelling after ASDH**^[^22^]^**. Another study on aquaporin-4 (AQP4), the principal water channel in the central nervous system, is also associated with variable outcomes after TBI, supporting the link between genotype and the development of brain edema**^[^23^]^**. Therapies targeting SUR1 and AQP4 could improve outcomes and help guide treatment decisions.

## Advanced diagnostics and imaging (500 words)

ASDH ranks among the most urgent neurosurgical emergencies, carrying not only a high risk of mortality but also the potential for lasting neurological disability. Traditionally, conventional CT has been the first-line tool for diagnosis and decision-making. However, the landscape is shifting. Advances in radiomics, perfusion and diffusion imaging, AI, and portable point-of-care technologies are transforming how clinicians assess patients, stratify risks, and guide treatment. Radiomics makes it possible to go beyond the traditional visual interpretation of CT and magnetic resonance imaging (MRI), extracting large volumes of quantitative information from routine scans. In the context of ASDH, these radiomic “signatures” can capture subtle features invisible to the naked eye and have shown promising associations with clinically relevant outcomes in early studies – for example, predicting the likelihood of postoperative epileptic seizures with notable accuracy**^[^24^]^**. It is important to note that many of these imaging advances are derived from broader TBI populations and may not yet be fully validated specifically in ASDH cohorts.

When these imaging-derived patterns are combined with circulating biomarkers such as GFAP, UCH-L1, and S100B, they create the foundation for radiogenomics, a field that integrates imaging and molecular data. This convergence not only refines prognostic models but also opens the door to more individualized and reliable predictions of patient trajectories**^[^25,26^]^**.

In neuroimaging, CT perfusion imaging (CTP) provides important information on CBF, cerebral blood volume, and mean transit time, which helps identify the ischemic penumbra and can guide early surgical intervention**^[^27,28^]^**. Clinical studies in neurotrauma referral centers have demonstrated that CTP can predict in-hospital mortality in patients with severe TBI with near-perfect specificity**^[^29^]^**. Diffusion-weighted imaging and diffusion tensor imaging (DWI/DTI), on the other hand, reveal diffuse axonal injury and traumatic microinfarcts that remain invisible on conventional CT, showing strong correlations with long-term neurological outcomes**^[^30,31^]^**.

Susceptibility-weighted imaging (SWI) enables the early detection of microhemorrhages associated with trauma, which are often underestimated by conventional CT**^[^32,33^]^**. High-definition fiber tracking (HDFT), in turn, enables the detailed reconstruction of disrupted white matter tracts, with significant implications for prognosis and surgical planning**^[^34^]^**.

Deep learning–based AI models are increasingly being explored for application to CT and MRI scans to predict intracranial hypertension (ICP), the need for decompressive surgery, and functional outcomes**^[^35,36^]^**. The multicenter CENTER-TBI study demonstrated that predictive algorithms integrating clinical and imaging data achieve higher accuracy in forecasting mortality and functional disability compared with traditional scoring systems**^[^37,38^]^**.

In LMICs, limited resources have driven the adoption of portable technologies such as mobile CT, transcranial ultrasound, and embedded AI platforms. These tools enable bedside ICP monitoring and aid rapid decision-making, reducing costs and diagnostic delays**^[^10,39–41^]^**. The future of diagnostic evaluation in ADHD will be defined by multimodal integration, combining radiomics analysis, advanced perfusion imaging, microstructural diffusion, SWI/HDFT techniques, and AI-based algorithms. This convergence is expected to increase prognostic accuracy, improve patient selection for decompressive surgery, and enable personalized postoperative follow-up. Furthermore, the comparative surgical outcomes of DC, craniotomy with bone flap replacement, and cranioplasty (Table 1) illustrate how the integration of imaging, biomarkers, and technologies can redefine neurosurgical practice in HSDA**^[^42^]^**.

**Table 1:** Comparative outcomes of DC, CO, and cranioplasty in ASDH (based on mixed evidence from randomized trials, observational studies, and broader TBI literature).

Procedure	Mortality	Functional outcome (GOS/GOS-E)	Complications (hydrocephalus, SSI, seizures)	References
Decompressive craniectomy (DC)	Reduced mortality in selected patients (~30–40%)	Limited functional improvement; high survival with disability	Hydrocephalus, epilepsy, and surgical site infection	**^[^37,38^]^**
Craniotomy with bone flap replacement	Comparable mortality in less severe cases	Potential for better functional recovery (GOS-E > 4)	Lower risk of late complications	**^[^10,37^]^**
Cranioplasty (post-DC)	Not applicable as an initial intervention	Associated with delayed cognitive and motor improvement	Implant infection, bone flap resorption	**^[^38,39^]^**

Note: Data presented in Table 1 represent general trends derived from heterogeneous sources including randomized trials, observational cohorts, and broader TBI literature. These comparisons should be interpreted as illustrative rather than definitive.

## Surgical continuum: DC, CO, and cranioplasty

The surgical management of ASDH extends far beyond the initial evacuation and decompression. The decision to perform a DC or a CO initiates a complex, multi-stage patient journey fraught with potential complications and critical follow-on decisions, most notably cranioplasty. This surgical continuum represents a challenging pathway where initial life-saving interventions must be balanced against long-term functional outcomes and the risks of subsequent procedures.

### DC vs CO: survival vs long-term function

The fundamental surgical dilemma in ASDH is choosing between a CO and a DC. While DC is unequivocally life-saving in scenarios of refractory intracranial hypertension, its impact on long-term functional recovery is highly contentious. The landmark RESCUE-ASDH trial randomized 408 patients with ASDH and TBI to either DC or CO. The primary finding was that DC was associated with a significantly lower mortality rate at 6 months compared to CO, 26.8% and 36.4%, respectively**^[^43^]^**. However, this survival benefit came at a cost, as a higher proportion of survivors in the DC group experienced an unfavorable functional outcome [Glasgow Outcome Scale-Extended (GOSE) score of 1–4)**^[^43^]^**. This starkly illustrates the ethical and clinical challenge, i.e., prioritizing survival against independent function.

Data from large observational studies like CENTER-TBI further contextualize this dilemma. While not a randomized surgical trial, its comprehensive data highlight that patients undergoing DC are often younger and have more severe injuries, making direct comparisons difficult**^[^44^]^**. The consensus is shifting toward a more nuanced approach, reserving DC for the most severe cases where medical management of ICP has failed, rather than as a first-line intervention for all ASDH cases. The goal is to avoid the long-term sequelae associated with the decompressed state whenever possible.

### Complications associated with decompression

Leaving the cranial vault open predisposes patients to a host of specific complications. Hydrocephalus is a frequent concern, as the loss of normal CSF dynamics and intracranial compliance can disrupt flow and reabsorption. Surgical site infections and meningitis are persistent risks, compounded by the presence of a large skull defect. Post-traumatic epilepsy is significantly more common after DC than CO, with the cortical scar and gliosis acting as a persistent seizure focus**^[^45^]^**. A particularly dramatic complication is paradoxical herniation, where a sunken skin flap above the defect creates a negative pressure gradient, sucking the brain downward through the dural opening, especially after lumbar puncture or even spontaneously. These complications collectively contribute to the high morbidity observed in DC survivors.

### Cranioplasty as therapy: beyond cosmetics

Cranioplasty, the surgical repair of the skull defect, is far more than a cosmetic procedure. It is a crucial therapeutic step in neurological rehabilitation. Restoring the integrity of the cranium normalizes ICP dynamics and CSF flow. It is crucial in re-establishing normal CPP, thereby improving CBF to the previously decompressed and often hypoperfused hemisphere**^[^46^]^**. Emerging evidence also suggests that cranioplasty may facilitate the function of the glymphatic system, which is dependent on arterial pulsations that are aberrant in the decompressed state. Clinically, this physiological restoration often translates to tangible neurological improvements, with numerous studies documenting significant enhancements in cognitive function, motor power, and overall functional status following cranioplasty**^[^46^]^**.

### Autologous vs alloplastic material innovations

The choice of material for cranioplasty is a key consideration. The autologous bone flap, stored after DC, is the gold standard due to its perfect fit, biocompatibility, and cost-effectiveness. However, its use is hampered by high resorption rates, the risk of infection from prior contamination, and fragility. Alloplastic materials have thus gained prominence. Polymethylmethacrylate is a longstanding, cost-effective option, though it lacks osteointegration. Polyetheretherketone is a premium synthetic implant celebrated for its excellent biomechanical properties, radiolucency, and customizability via prefabrication from patient CT scans. Titanium mesh is robust and osteointegrating but can conduct heat and cold and is more challenging to shape intraoperatively. The horizon of materials science points toward 3D-bioprinted scaffolds. These constructs aim to be more than inert implants; they are designed with bioactive materials and porous architectures that encourage native bone ingrowth, vascularization, and ultimately, true cranial regeneration**^[^47^]^**.

### Timing debate: early vs delayed cranioplasty

The optimal timing for cranioplasty remains a subject of active debate, balancing the benefits of early neurological recovery against the risks of operating on a recently injured brain. Proponents of early cranioplasty (typically defined as within 3 months of DC) argue that it accelerates neurological improvement, reduces complications from the skull defect, and shortens the overall recovery timeline. Conversely, those advocating for a delayed approach cite a potentially higher risk of infection if performed while the brain is still inflamed, swollen, or the scalp is poorly perfused. A large meta-analysis suggested that early cranioplasty may be associated with a higher incidence of postoperative seizures but similar rates of infection compared to later surgery**^[^48^]^**. The trend is moving toward earlier intervention when the patient’s clinical condition is stable and the scalp is supple, recognizing cranioplasty as a vital step in neurological convalescence rather than an elective procedure to be indefinitely delayed.

## Emerging technologies

Magnetic resonance (MR)-guided focused ultrasound is the center of interest and development in minimal and noninvasive therapies. Being explored primarily in oncological neurosurgery and movement disorders, its application has now been growing and is also being seen in life-threatening (TBIs), particularly ASDH. MR-guided focused ultrasound (MRgFUS) surgery is an emerging technology that permits a highly concentrated focal point of ultrasound energy to be deposited at a precise target deep within the brain without an incision or CO. With appropriate ultrasound parameters, it has been shown that MRgFUS can effectively liquefy large-volume blood clots through the human calvaria, provide real-time procedural control, and ensure accurate outcome assessment. In addition, ongoing research suggests that MRgFUS has potential in regulating cerebral blood flow and ICP. Although still in its initial stages, this technology represents a promising but investigational shift toward noninvasive neurosurgical intervention**^[^49–51^]^**.

Intraoperative imaging and navigation, already showcasing its potential in other neurosurgical domains, has now begun to influence trauma surgery. Real-time intraoperative CT, ultrasound, and precision-based perfusion imaging allow the surgeon to confirm the extent of hematoma evacuation, detect contralateral or remote lesions, brain swelling, re-bleeds, and possible evolution of a primary head injury, leading to early and effective treatment. Its routine utilization permits surgical performance assessment, provides the chance for immediate re-aspiration, targets an ideal residual hematoma volume, and reduces secondary revision rates. Therefore, being a fast, easy, and low-cost technique, it is a game-changing approach, offering surgeons valuable insights and providing clarity to resolve many of the unanswered questions**^[^52–55^]^**.

Personalized implants – 3D-printed bioengineered implants – are yet another promising approach in the field of ASDH and TBI. Following decompressive procedures, the art of cranioplasty focuses not only on cosmesis and biomechanical strength but also on neurocognitive recovery and ICP dynamics. Advancements in these approaches have enabled patient-specific implants to reduce infection risk, bone flap resorption, and may even promote osteointegration and neuronal repair. They have been shown to overcome a major physiological challenge: the restoration of ICP equilibrium, thereby improving cerebral blood flow, cerebral perfusion, and potentially enhancing neurological outcomes. Being a niche and emerging application in the area of ASDH and TBI, early clinical outcomes have demonstrated success and full potential is yet to be explored and systematically validated**^[^56–59^]^**. ASDH surgery is shifting from simple evacuation to integrated, tech-based care.

## Global and ethical perspectives

### Variation in surgical strategies: high-income countries vs LMICs

A survey by M. Mohan, which collected data from neurosurgeons spanning 60 countries, shows that DC is the first-choice procedure worldwide, especially for post-TBI cerebral decompression due to high ICP, although the first-choice approach changes based on circumstances among countries**^[^60,61^]^**.

LMICs mostly choose decompressive craniotomy (DCO) due to the high price and low accessibility of neurosurgery, as well as limited ICP monitoring. Hinge craniotomy (HC) is an alternative in LMICs as it reduces the need for a secondary cranioplasty surgery**^[^60,61^]^**. Another surgical procedure in resource-limited countries is burr-hole surgery, as it is safe and impactful on survival and morbidity, especially when DC or DCO cannot be immediately performed. However, it is uncommon and not standardized for hematoma evacuation**^[^62,63^]^**.

High-income countries (HICs) prefer DCO primarily, such as the United States and several European countries. However, DC is being performed in cases of bone flap defects, a high possibility of brain swelling predicted during an operation, and refractory intracranial hypertension. ICP monitoring and intraoperative findings help in deciding which surgical procedure needs to be performed**^[^12,61,64^]^**.

### Cost-effectiveness of DC vs CO vs staged approaches

According to randomized economic evidence, DC is less affordable compared to DCO due to the subsequent additional cranioplasty surgery needed to replace the bone flap, even if it is autologous, associated complications, and longer ICU/hospitalization**^[^60,65^]^**.

Post-DC cranioplasty costs change based on the materials used during the operation, and this needs to be considered in LMICs and even in a few HICs where they are funded by their governments and may lead surgeons to choose other options rather than DC**^[^65^]^**.

As an alternative to DC, HC is a cost-effective decompression technique that has a reducing effect on brain swelling and does not require a second surgery**^[^66^]^**. While staged/hybrid approaches (starting with DCO followed by DC in case of brain swelling) are a combined method that requires continuous monitoring and leads to short-term high-costs, yet long-term low-costs**^[^63,66^]^**.

Burr-hole surgery and drainage are other alternatives in resource-limited settings for immediate stabilization while being an affordable approach. However, it has long-term risks and additional operation needs due to ineffective hematoma evacuation, which can lead to further expenses**^[^63^]^**.

Another option is cisternostomy, where the basal cisterns are opened, which could reduce swelling of the brain and ICP. However, this requires more microsurgical tools and microscopes, making it a less affordable option in resource-limited conditions**^[^66^]^**.

### Ethical dilemmas: survival vs quality of life, decision-making capacity

Moral conflicts when dealing with ASDH regarding biological survival vs quality of life are always seen among neurosurgeons**^[^67^]^**.

Early intervention provides low mortality rates, yet it cannot prevent disabilities. Retrospective studies regarding postoperative functional recovery, measured by the GOS-E, and well-being, measured by the questionnaire Quality of Life after Brain Injury (QOLIBRI), show favorable but still lower-than-expected results**^[^68^]^**. A result of another study on elderly patients shows that early surgery yields survival but poor quality of life, whereas a delayed operation or a conservative approach provides better functional outcomes**^[^69^]^**.

Since questions on therapeutic success exist, researchers are having disagreements on whether surgeons should withhold surgery due to the low possibility of future well-being or perform the operation just to save lives, even if it results in tragic postoperative suffering**^[^70^]^**.

While making a decision, multidisciplinary consideration of legal and ethical boundaries that emphasizes certain clinical observations, being transparent with the family with consent/reason documentation, acting for the benefit of the patient, respecting their values and dignity, and avoiding harm is crucial**^[^67,70^]^**.

## Future directions and research agenda

The management of ASDH is entering a transformative era, driven by advances in precision neurosurgery, digital health, and regenerative neurotherapies. Traditional decision-making, largely guided by clinical severity and imaging, is increasingly complemented by biomarker-driven algorithms that stratify patients for optimal surgical intervention. Emerging molecular and imaging biomarkers – including markers of coagulation, neuroinflammation, and neuronal injury – offer the potential to predict hematoma progression, ICP dynamics, and postoperative recovery trajectories, enabling personalized surgical planning**^[^7,71^]^**.

Integration of AI with multicenter registries further augments this precision approach. Platforms such as CENTER-TBI and TRACK-TBI, alongside nascent LMIC registries, provide rich longitudinal datasets that facilitate machine-learning models to identify predictors of outcome, refine operative timing, and optimize postoperative care pathways. AI-enabled decision support can also aid in real-time triage, imaging interpretation, and risk stratification, addressing inter-surgeon variability and improving consistency of care across diverse healthcare settings**^[^72–74^]^**.

Concurrently, regenerative cranioplasty and neurorestoration represent an emerging frontier in ASDH management. Biocompatible scaffolds, autologous stem-cell therapies, and 3D-printed cranial implants hold promise for improved structural and functional recovery, potentially mitigating complications associated with conventional cranioplasty and enhancing neurocognitive rehabilitation**^[^75,76^]^**. Early translational studies suggest that integrating these technologies with precision-guided surgical strategies could extend the therapeutic window and optimize long-term neurological outcomes.

Future research should prioritize prospective, multicenter trials that evaluate integrated precision-neurosurgery workflows, AI-assisted decision-making, and regenerative interventions, with attention to equity across resource settings. Emphasizing standardized outcome measures – including 12-month functional independence, quality of life, and caregiver burden – will be essential for assessing true clinical impact and facilitating global applicability. Collectively, these innovations promise a paradigm shift from reactive surgery to proactive, individualized care, aligning operative strategy with patient biology, technology, and system-level equity. Future research should prioritize ASDH-specific prospective datasets rather than relying predominantly on extrapolated TBI data.

## Conclusion

The management of ASDH is a continuous, patient-specific surgical process. Advances in biomarkers, imaging techniques, and surgical strategies, alongside the patient’s biological risk profile, tailor surgical decision-making. Inflammatory markers such as IL-6 and CRP indicate inflammation; coagulation markers such as D-dimer, fibrinogen, and low platelet count indicate the risk of hematoma expansion; neurodegeneration markers such as GFAP, NSE, and tau indicate the extent of brain damage. These biomarkers may enable risk-adapted strategies and support clinical decision-making; however, their role in directly determining surgical choice requires further prospective validation. Standardizing biomarker use may improve decision-making that ultimately benefits long-term functional independence, quality of life, and caregiver burden.

Advances in radiomics, integrating imaging and biomarker profiles beyond the traditional visual interpretation of CT and MRI, improve risk stratification and clinically relevant outcomes. CTP identifies ischemic penumbra, DWI/DTI reveals diffuse axonal injury and traumatic microinfarcts, SWI enables early detection of microhemorrhages associated with trauma, and deep learning-based AI models now augment the detection of ICP and the need for decompressive surgery. Combining biomarkers and radiologic findings helps assess brain injury and, therefore, guide surgical choices. Low-biomarker-risk patients with favorable CT findings are candidates for CO. Conversely, high-biomarker-risk patients with signs of swelling on CT are candidates for DC.

On surgical timing, earlier intervention is better once clinical stability and scalp integrity are achieved. Early cranioplasty has been associated with improved neurological recovery and shorter rehabilitation times. Postoperative seizures are significantly more common after DC than CO.

Overall, the integration of biology, technology, and global health system factors allows personalized, proactive management of ASDH across the surgical continuum. Importantly, many biomarker-driven and technology-integrated approaches remain in early or translational stages and require robust prospective validation before routine clinical implementation.

Lastly, this narrative review adheres to the TITAN guidelines**^[^8^]^**. During the preparation of this manuscript, generative AI tools (DeepSeek, DeepSeek Inc., June 2025 version: temperature parameter = 0.7) were used only as auxiliary aids to assist in formatting, standardization, organization of background information, and language consistency checks. Furthermore, all AI-assisted text was thoroughly reviewed, edited, and refined by the authors. The conceptualization, literature interpretation, synthesis of ideas, and manuscript writing were performed entirely by the authors, ensuring the academic rigor, accuracy, and originality of the review.

## Data Availability

All data analyzed in this study are derived from previously published articles and publicly available sources, as cited in the reference list.

## Research registration unique identifying number (UIN)

Unique Identification Number (UIN): Not applicable.

This study is a narrative review of previously published literature and did not require prospective registration.
